# Real-world treatment patterns of OTX-101 ophthalmic solution, cyclosporine ophthalmic emulsion, and lifitegrast ophthalmic solution in patients with dry eye disease: a retrospective analysis

**DOI:** 10.1186/s12886-023-03174-y

**Published:** 2023-11-02

**Authors:** Paul Karpecki, Victoria Barghout, Brad Schenkel, Lynn Huynh, Anamika Khanal, Brittany Mitchell, Mihran Yenikomshian, Enrico Zanardo, Cynthia Matossian

**Affiliations:** 1 Kentucky Eye Institute, University of Pikeville Kentucky College of Optometry, Lexington, KY USA; 2 VEB HealthCare, Morristown, NJ USA; 3 Sun Pharmaceutical Industries, Inc., Princeton, NJ USA; 4 Analysis Group, Inc., Boston, MA USA; 5 Analysis Group, Inc., Denver, CO USA; 6 CM Associates, LLC, New Hope, PA USA

**Keywords:** Cyclosporine A, Discontinuation, Keratoconjunctivitis sicca, Persistence

## Abstract

**Background:**

Dry eye disease (DED) is a disorder characterized by loss of tear film homeostasis that causes ocular surface inflammation and damage. The incidence of DED increases with age. Cyclosporine ophthalmic solution 0.09% (CEQUA^®^; OTX-101), cyclosporine ophthalmic emulsion 0.05% (Restasis^®^; CsA), and lifitegrast ophthalmic solution 5% (Xiidra^®^; LFT) are anti-inflammatory agents indicated for DED. This analysis compared treatment patterns in patients with DED receiving OTX-101, CsA, or LFT.

**Methods:**

This real-world, retrospective, longitudinal cohort study utilized Symphony Health Integrated Dataverse claims from July 2019 to June 2021. The dataset included all patients with OTX-101 claims and patients with CsA or LFT claims randomly selected 2:1 to OTX-101. Patients were sorted into 3 cohorts based on index treatment. Index date was that of first treatment claim, and follow-up period was from index date to end of clinical activity or data availability. Time to treatment discontinuation (TTD), probability of discontinuation, and treatment persistence were assessed for OTX-101 vs. CsA, then OTX-101 vs. LFT. Subgroup analysis was performed based on age and prior DED treatment. Kaplan-Meier analysis and log-rank test were used to examine TTD. A logistic model evaluated association between index treatment and discontinuation. Unadjusted and adjusted odds ratios, 95% confidence intervals, and *P*-values were reported, with statistically significant associations based on *P*-values < 0.05.

**Results:**

Overall, 7102 patients (OTX-101 *n* = 1846; CsA *n* = 2248; LFT *n* = 3008) were eligible. Median TTD was 354 days for patients receiving OTX-101 vs. 241 days for CsA and 269 days for LFT. Log-rank test indicated TTD was significantly longer for patients on OTX-101 vs. CsA (*P* = 0.033). Patients on CsA were 35% more likely to discontinue treatment than patients on OTX-101; OTX-101 and LFT groups had similar discontinuation rates. After 360 days, 49.8% of patients receiving OTX-101 remained on treatment vs. 39.4% of patients on CsA (*P* = 0.036) and 44.0% of patients on LFT (*P* = 0.854).

**Conclusions:**

Patients receiving OTX-101 remained on treatment significantly longer and were significantly less likely to discontinue treatment than patients on CsA. Older patients remained on OTX-101 significantly longer than CsA. These findings highlight treatment pattern differences in patients with DED receiving these anti-inflammatory agents.

**Supplementary Information:**

The online version contains supplementary material available at 10.1186/s12886-023-03174-y.

## Background

 Dry eye disease (DED) is a multifactorial, inflammatory disease of the ocular surface characterized by loss of tear film homeostasis, which affects an estimated 5.3% of the overall US population [1, 2]. The prevalence of DED in the US increases with age, with an estimated incidence of 7.8% in individuals aged 68 years or older [3]. DED may develop secondary to dysfunction of any of the ocular structures that produce tear film components, including the lacrimal glands, meibomian glands, or conjunctival epithelium [4]. Dysfunction of these structures may result in decreased tear production and/or increased tear evaporation, which lead to tear film hyperosmolarity and instability and subject the ocular surface to desiccating stress [1, 4].

Desiccation of the ocular surface triggers initiation of a self-perpetuating cycle of ocular surface inflammation and damage [1, 4, 5]. Ocular surface inflammation may cause meibomian gland dysfunction, lacrimal gland epithelial cell destruction and dysfunction, and/or apoptosis of corneal and conjunctival epithelial and goblet cells, leading to further tear film deficiencies [1, 4]. Over time, progressive ocular surface damage secondary to DED can impair visual function, and the chronic ocular discomfort symptoms associated with DED may decrease patients’ quality of life [4, 6]. Thus, the use of anti-inflammatory agents that target the underlying disease process has become a mainstay of DED management [5, 7].

Common anti-inflammatory medications utilized for DED treatment include corticosteroids, cyclosporine A, and lifitegrast [7]. As chronic topical corticosteroid use is associated with ocular adverse effects, including increased intraocular pressure, worsening or de novo cataract development, and opportunistic infections, topical cyclosporine A and lifitegrast are often used for long-term management of DED [7, 8].

Cyclosporine is a calcineurin inhibitor immunosuppressant thought to act as a partial immunomodulator in patients with DED [9, 10]. Cyclosporine ophthalmic emulsion 0.05% (Restasis®; CsA) was the first cyclosporine A eyedrop approved by the US Food and Drug Administration (FDA) in 2003. It is indicated to increase tear production in patients whose tear production is presumed to be suppressed due to ocular inflammation associated with DED [10, 11]. Cyclosporine ophthalmic solution 0.09% (CEQUA®; OTX-101), which was developed as a novel nanomicellar solution in contrast to the oil-in-water emulsion of CsA, was approved by the FDA in 2018 to increase tear production in patients with DED [9, 11]. Lifitegrast is a lymphocyte function-associated antigen-1 (LFA-1) antagonist that blocks the interaction of LFA-1 with intercellular adhesion molecule-1 (ICAM-1), preventing T-cell activation and migration to target tissues. ICAM-1 may be overexpressed in the cornea and conjunctiva in patients with DED [12]. Lifitegrast ophthalmic solution 5% (Xiidra®; LFT), approved by the FDA in 2016, is indicated for the treatment of the signs and symptoms of DED [12].

Although each of these anti-inflammatory therapeutics has been found effective and safe for DED treatment in multiple pivotal clinical trials [13–20], limited comparative data are available regarding their use in real-world settings. Therefore, the objective of this study was to compare treatment patterns of OTX-101, CsA, and LFT among real-world patients with DED.

## Methods

### Study design and data source

**Fig. 1:**
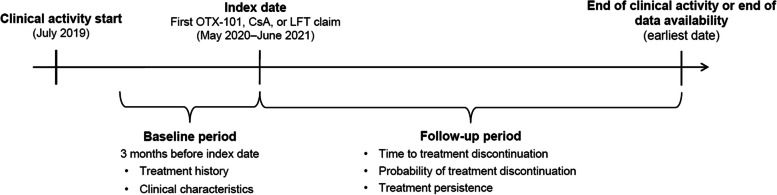
Study design. CsA, cyclosporine ophthalmic emulsion 0.05%; LFT, lifitegrast ophthalmic solution 5%; OTX-101, cyclosporine ophthalmic solution 0.09%

This was a real-world, retrospective, longitudinal cohort study; the study design is depicted in Fig. 1. The study utilized claims data from the Symphony Health Integrated Dataverse (IDV; Symphony Health, Blue Bell, PA) spanning 24 months from July 2019 to June 2021. The IDV is a nationally representative, provider-based claims database including claims submitted to all payer types (commercial plans, Medicare, cash, assistance programs, Medicaid, etc.) that covers approximately 75% of the US population (280 million lives) annually. The IDV captures approximately 70% of US retail and specialty pharmacy claims and 60% of pharmacy mail orders, and covers approximately 55% of professional medical claims and 30% of institutional medical claims.

The study’s index date was the date of treatment initiation (first treatment claim), and the index treatment was OTX-101 for patients with at least 1 OTX-101 claim, or the earlier of CsA or LFT for patients without an OTX-101 claim. The baseline period was the 3 months prior to the index date when patient demographics and clinical characteristics were assessed. The follow-up period was from the index date to either the end of clinical activity (defined as the date of the last encounter) or the end of data availability, whichever occurred first. This study received a waiver of authorization for use and disclosure of protected health information from, and was determined Institutional Review Board (IRB) exempt by, WCG™ IRB. The research was conducted in accordance with the Declaration of Helsinki.

The dataset included all patients with an OTX-101 claim and patients with CsA or LFT claims randomly selected 2:1 relative to OTX-101 patients. All database records are de-identified and fully compliant with US patient confidentiality requirements, including the Health Insurance Portability and Accountability Act of 1996.

### Study population

International Classification of Diseases (ICD) codes were used to identify patients with DED to be included in the study population. Primary diagnosis ICD-9-CM and ICD-10-CM codes included keratoconjunctivitis sicca; tear film insufficiency, unspecified; exposure keratoconjunctivitis; neurotrophic keratoconjunctivitis; superficial keratoconjunctivitis; punctate keratitis; conjunctival xerosis; sicca syndrome; and ocular pain (Supplemental Table 1). Eligible patients were at least 18 years old at the index date, with a first claim for OTX-101, CsA, or LFT from May 2020 to June 2021. Patients were required to have at least 2 documented medical claims before or on the index date and at least 1 additional claim for index treatment within the first 4 months after the index date. Additionally, patients were required to have at least 1 diagnosis of DED between July 2019 and June 2021 and evidence of clinical activity during the baseline period and within 1 year after the index date. Patient identification was based on treatment initiation in or after May 2020 in order to minimize the impact of COVID-19 shutdowns. No specific exclusion criteria were applied in this study.

Three patient cohorts were established based on index treatment. The OTX-101 cohort included patients with a first OTX-101 claim from May 2020 to July 2021. The CsA cohort included patients who did not receive OTX-101 from October 2019 to July 2021, had a first CsA claim after May 2020, and had no LFT claims between May 2020 and CsA initiation. The LFT cohort included patients who did not receive OTX-101 from October 2019 to July 2021, had a first LFT claim after May 2020, and had no CsA claims between May 2020 and LFT initiation.

### Assessments

#### Patient characteristics

Patient characteristics were assessed during the baseline period. Patient demographics included patient age, sex, and geographic region. Clinical characteristics included type of insurance plan, year of index date, and length of follow-up period. Disease characteristics included DED diagnosis, year of first DED diagnosis, comorbidities (scored using the Quan-Charlson Comorbidity Index [Quan-CCI]), other comorbidities not considered in the Quan-CCI, and medications received during the baseline period.

#### Outcome measures

Study endpoints included time to treatment discontinuation (TTD), probability of treatment discontinuation, and treatment persistence for OTX-101, CsA, and LFT. Treatment discontinuation was defined as a period of more than 120 days between prescription claims or between the last prescription claim and the end of continuous clinical activity or data availability. TTD was the time from treatment initiation to the onset of discontinuation. Treatment persistence evaluated the percentage of patients remaining on each treatment at various times after the index date. Analyses first compared OTX-101 vs. CsA, and then OTX-101 vs. LFT. Subgroup analyses were also conducted based on age and DED treatments received prior to index date.

### Statistical analysis

Patient demographics, disease, and clinical characteristics described during the baseline period were summarized as means, standard deviations (SDs), interquartile ranges (IQRs), and medians for continuous variables, and as frequencies and percentages for categorical variables. Treatment pattern variables were summarized as means, SDs, IQRs, and medians for continuous variables, and as frequencies and percentages for categorical variables. Kaplan-Meier analysis and log-rank test were used to examine TTD. Kaplan-Meier curves were prepared for the described subgroups, and medians and corresponding 95% confidence intervals (CIs) were calculated. A logistic model was used to assess the association between index treatment and treatment discontinuation. Both unadjusted and adjusted odds ratios (ORs), 95% CIs, and *P*-values were reported, and statistically significant associations were determined based on *P*-values less than 0.05.

## Results

### Patient demographics

**Fig. 2:**
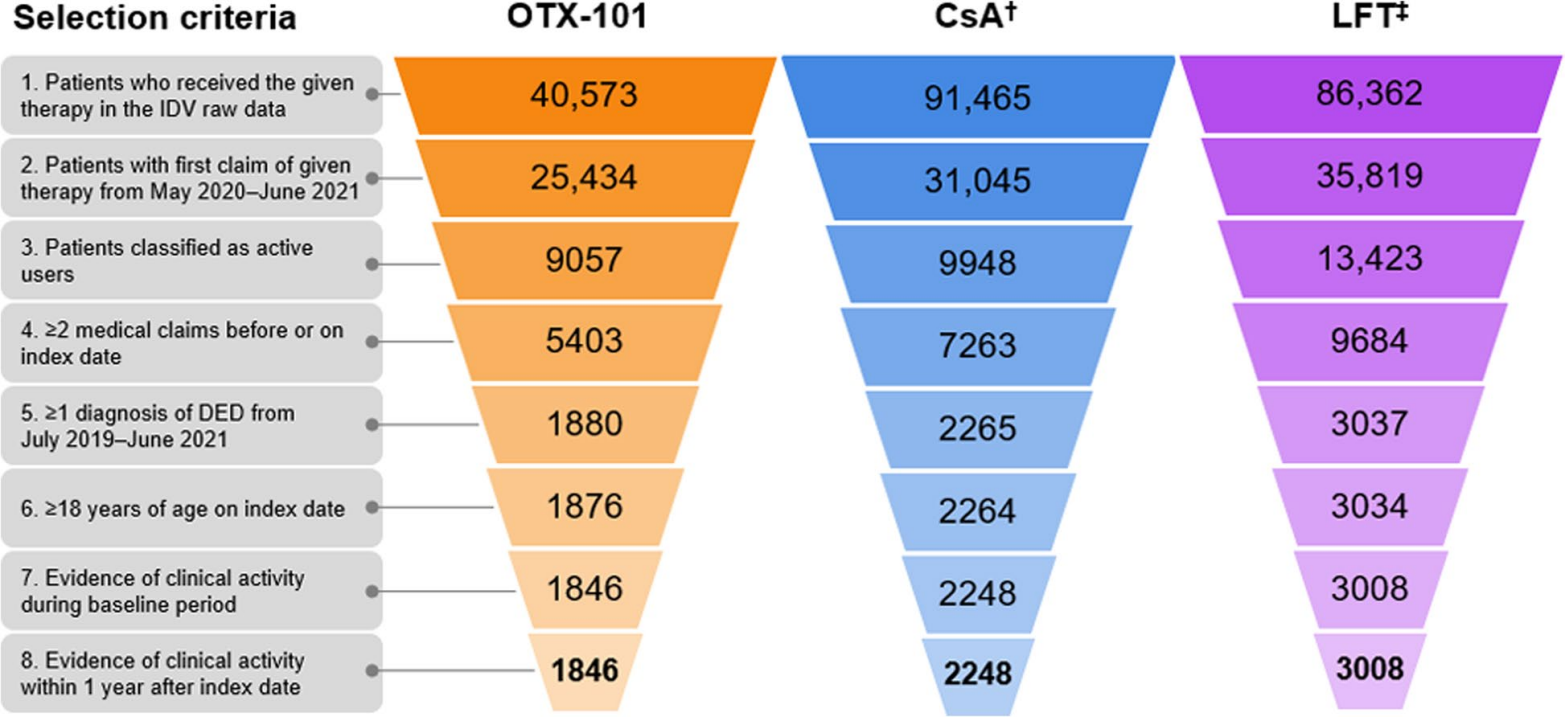
Study population selection. Note: Listed numerical values indicate the number of patients meeting the specified selection criterion for each step. ^†^CsA cohort excludes patients who received OTX-101 at any point (*n* = 2971) or received LFT before CsA between May 2020 and June 2021 (*n* = 1465). ^‡^LFT cohort excludes patients who received OTX-101 at any point (*n* = 2380) or received CsA before LFT between May 2020 and June 2021 (*n* = 1753). CsA, cyclosporine ophthalmic emulsion 0.05%; DED, dry eye disease; IDV, Symphony Health Integrated Dataverse; LFT, lifitegrast ophthalmic solution 5%; OTX-101, cyclosporine ophthalmic solution 0.09%

In the Symphony IDV raw data, 40,573 total patients were identified as having received OTX-101, and random samples of 91,465 patients who received CsA and 86,362 patients who received LFT were drawn. Following implementation of the inclusion criteria, 7102 total patients were included in the final analysis: 1846 patients in the OTX-101 cohort, 2248 patients in the CsA cohort, and 3008 patients in the LFT cohort (Fig. 2). Among the 3 treatment groups, mean ages ranged from 61–65 years old, and over 83% of patients were female. The average length of follow-up ranged from 6.8–7.6 months (Table 1). Tear film insufficiency was the most common DED diagnosis, noted in over 70% of patients in all cohorts, and most patients had Quan-CCI scores of 0, indicating no major comorbidities. Notably, high proportions of patients in all cohorts were taking antidepressant or anti-anxiety medications in the 3 months before the index date (Table 2).

**Table 1:** Patient demographics and baseline characteristics

	OTX-101 *n* = 1846	CsA *n* = 2248	LFT *n* = 3008
**Age at index date**, years			
Mean ± SD	60.6 ± 13.0	64.6 ± 11.9	62.4 ± 11.9
Median (IQR)	62.0 (53.0, 71.0)	66.0 (58.0, 74.0)	63.0 (55.0, 72.0)
**Sex**, n (%)			
Female	1539 (83.4)	1935 (86.1)	2555 (84.9)
Male	307 (16.6)	313 (13.9)	453 (15.1)
**Geographic region**, n (%)			
South	656 (35.5)	829 (36.9)	1061 (35.3)
Northeast	477 (25.8)	659 (29.3)	945 (31.4)
West	425 (23.0)	314 (14.0)	418 (13.9)
Midwest	280 (15.2)	445 (19.8)	584 (19.4)
**DED diagnosis**, n (%)			
Tear film insufficiency, unspecified	1299 (70.4)	1639 (72.9)	2201 (73.2)
Keratoconjunctivitis sicca	570 (30.9)	630 (28.0)	778 (25.9)
Sicca syndrome, Sjögren	364 (19.7)	371 (16.5)	499 (16.6)
Ocular pain	114 (6.2)	102 (4.5)	144 (4.8)
Exposure keratoconjunctivitis	29 (1.6)	21 (0.9)	32 (1.1)
Neurotrophic keratoconjunctivitis	11 (0.6)	11 (0.5)	15 (0.5)
Punctate keratitis	3 (0.2)	3 (0.1)	2 (0.1)
Conjunctival xerosis	0	2 (0.1)	3 (0.1)
**Year of first DED diagnosis**, n (%)			
2019	723 (39.2)	782 (34.8)	1031 (34.3)
2020	861 (46.6)	1051 (46.8)	1383 (46.0)
2021	262 (14.2)	415 (18.5)	594 (19.7)
**Type of insurance plan**, n (%)			
Medicare	621 (33.6)	1244 (55.3)	1173 (39.0)
Commercial	603 (32.7)	589 (26.2)	766 (25.5)
Medicaid	232 (12.6)	268 (11.9)	316 (10.5)
Employer	155 (8.4)	196 (8.7)	394 (13.1)
Other	256 (13.9)	338 (15.0)	520 (17.3)
Unknown	183 (9.9)	203 (9.0)	413 (13.7)
**Year of index date**, n (%)			
2020	1157 (62.7)	1544 (68.7)	1726 (57.4)
2021	689 (37.3)	704 (31.3)	1282 (42.6)
**Follow-up period**, months			
Mean ± SD	6.8 ± 3.3	7.6 ± 3.5	6.8 ± 3.4
Median (IQR)	6.9 (4.2, 9.3)	7.8 (4.7, 10.5)	6.4 (3.9, 9.6)

*CsA* Cyclosporine ophthalmic emulsion 0.05%, *DED* Dry eye disease, *IQR* Interquartile range, *LFT* Lifitegrast ophthalmic solution 5%, *OTX-101* Cyclosporine ophthalmic solution 0.09%, *SD* Standard deviation

**Table 2:** Baseline patient comorbidities

	OTX-101 *n* = 1846	CsA *n* = 2248	LFT *n* = 3008
**Quan-CCI, n (%)**			
Mean ± SD	0.7 ± 1.4	1.2 ± 1.8	1.0 ± 1.7
Median (IQR)	0.0 (0.0, 1.0)	0.0 (0.0, 2.0)	0.0 (0.0, 2.0)
Score 0	1199 (65.0)	1128 (50.2)	1670 (55.5)
Score 1–2	513 (27.8)	729 (32.4)	926 (30.8)
Score 3–4	90 (4.9)	258 (11.5)	276 (9.2)
Score ≥ 5	44 (2.4)	133 (5.9)	136 (4.5)
**Other comorbidities**^**a**^, n (%)			
Eye-related comorbidities	449 (24.3)	466 (20.7)	652 (21.7)
Blepharitis	264 (14.3)	240 (10.7)	323 (10.7)
Visual disturbance	154 (8.3)	185 (8.2)	251 (8.3)
Allergic conjunctivitis	99 (5.4)	97 (4.3)	158 (5.3)
Major autoimmune disorders	52 (2.8)	96 (4.3)	85 (2.8)
Rheumatoid arthritis	52 (2.8)	96 (4.3)	85 (2.8)
Any other comorbidities	722 (39.1)	1146 (51.0)	1427 (47.4)
Thyroid disease	288 (15.6)	485 (21.6)	578 (19.2)
Anxiety	277 (15.0)	478 (21.3)	619 (20.6)
Depression	234 (12.7)	450 (20.0)	534 (17.8)
Fatigue	204 (11.1)	298 (13.3)	430 (14.3)
Menopause	121 (6.6)	163 (7.3)	235 (7.8)
Systemic lupus	47 (2.5)	68 (3.0)	112 (3.7)
**Medications**^**b**^, n (%)			
Antidepressant and anti-anxiety medication	861 (46.6)	1398 (62.2)	1768 (58.8)
Eye-related medication	823 (44.6)	560 (24.9)	829 (27.6)
CsA	293 (15.9)		241 (8.1)
LFT	236 (12.8)	117 (5.2)	
Other eye-related medication	474 (25.7)	473 (21.1)	646 (21.5)
High blood pressure medication	748 (40.5)	1201 (53.1)	1470 (48.9)
Hormone replacement therapy	473 (25.6)	707 (31.5)	839 (27.9)
Topical glaucoma medication	191 (10.3)	200 (8.9)	300 (10.1)
Topical allergy medication	162 (8.8)	220 (9.8)	290 (9.6)

*CsA* Cyclosporine ophthalmic emulsion 0.05%, *IQR* Interquartile range, *LFT* Lifitegrast ophthalmic solution 5%, *OTX-101* Cyclosporine ophthalmic solution 0.09%, *Quan-CCI* Quan-Charlson Comorbidity Index, *SD* Standard deviation

^a^Patient comorbidities any time prior to or on the index date were reported

^b^Medications received in the baseline period (3 months prior to the index date) were reported

### Time to treatment discontinuation

**Fig. 3:**
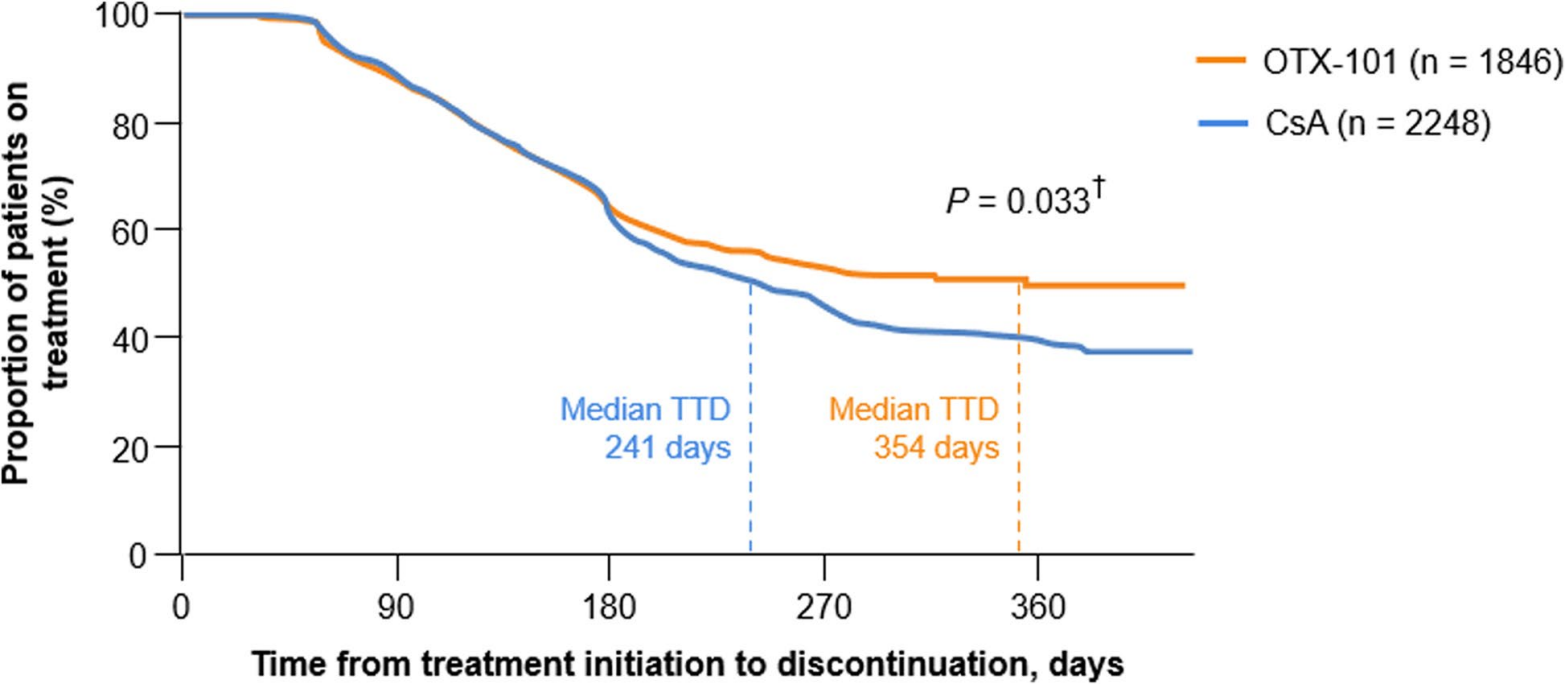
Kaplan-Meier survival curve for time to treatment discontinuation, OTX-101 vs. CsA. ^†^*P*-value refers to the difference between treatments based on the log-rank test. CsA, cyclosporine ophthalmic emulsion 0.05%; OTX-101, cyclosporine ophthalmic solution 0.09%; TTD, time to treatment discontinuation

**Fig. 4:**
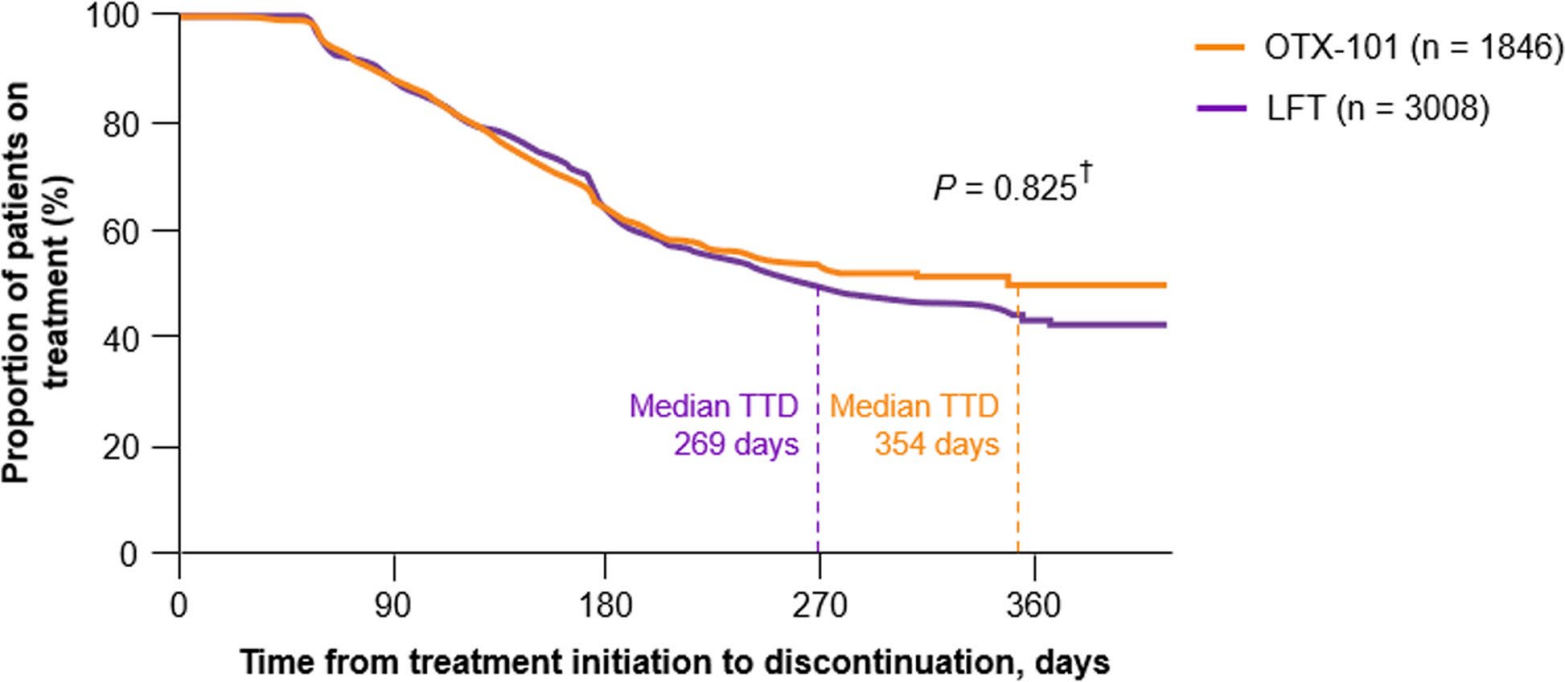
Kaplan-Meier survival curve for time to treatment discontinuation, OTX-101 vs. LFT. ^†^*P*-value refers to the difference between treatments based on the log-rank test. LFT, lifitegrast ophthalmic solution 5%; OTX-101, cyclosporine ophthalmic solution 0.09%; TTD, time to treatment discontinuation

 The median TTD was 354 days (95% CI, 268 to not estimable) for the OTX-101 cohort vs. 241 days (95% CI, 219 to 264) for the CsA cohort; the median treatment length for a patient receiving OTX-101 was 113 days (3.7 months) longer than that of a patient receiving CsA (Fig. 3). The median TTD was 269 days (95% CI, 248 to 303) for the LFT cohort (Fig. 4). Per the log-rank test, differences between cohorts were statistically significant for OTX-101 vs. CsA (*P* = 0.033) but not for OTX-101 vs. LFT (*P* = 0.825), though the median treatment length for a patient on OTX-101 was numerically longer than the median treatment length for a patient on LFT [21].

**Fig. 5:**
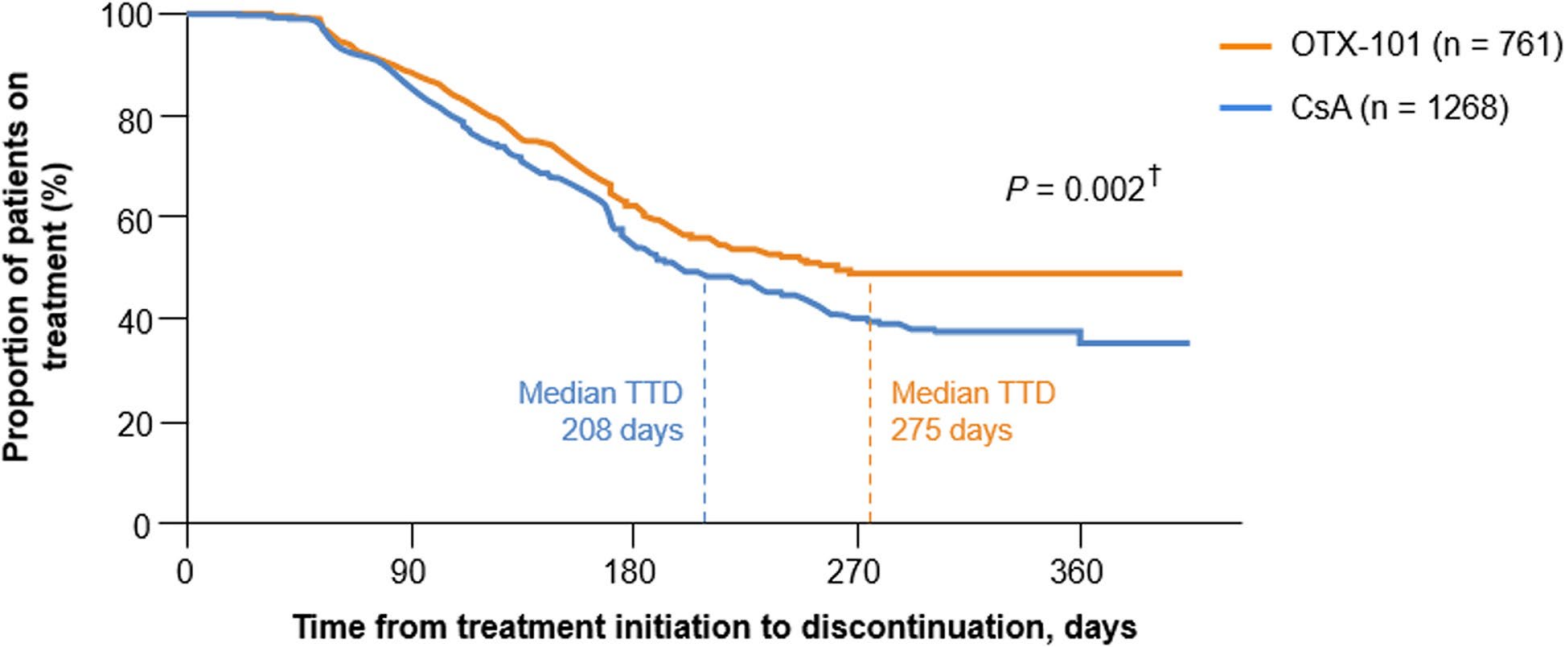
Kaplan-Meier survival curve, time to treatment discontinuation in patients > 64 years old, OTX-101 vs. CsA. ^†^*P*-value refers to the difference between treatments based on the log-rank test. CsA, cyclosporine ophthalmic emulsion 0.05%; OTX-101, cyclosporine ophthalmic solution 0.09%; TTD, time to treatment discontinuation

**Fig. 6:**
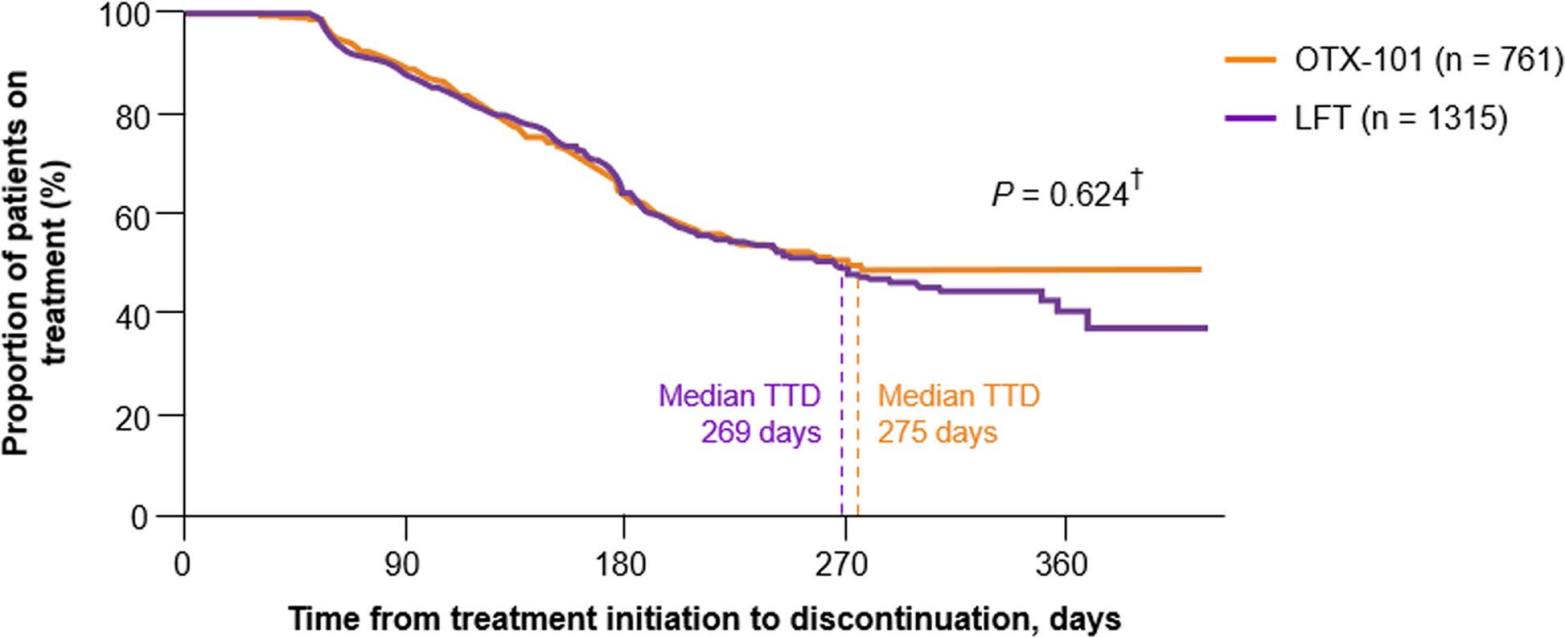
Kaplan-Meier survival curve, time to treatment discontinuation in patients > 64 years old, OTX-101 vs. LFT. ^†^*P*-value refers to the difference between treatments based on the log-rank test. LFT, lifitegrast ophthalmic solution 5%; OTX-101, cyclosporine ophthalmic solution 0.09%; TTD, time to treatment discontinuation

 In patients > 64 years of age (the median age of the study population at index date), those receiving OTX-101 stayed on treatment longer than those receiving CsA; median TTD was 275 days (95% CI, 225 to not estimable) for OTX-101 and 208 days (95% CI, 194 to 241) for CsA (Fig. 5). Per the log-rank test, the difference between cohorts was statistically significant (*P* = 0.002) [21]. The median treatment length for a patient on OTX-101 was 67 days (2.2 months) longer than that of a patient on CsA. Additionally, in this subgroup, median TTD was 269 days (95% CI, 235 to 313) for patients on LFT (Fig. 6). While patients receiving OTX-101 remained on treatment numerically longer than those receiving LFT, the log-rank test showed that the difference between cohorts was not statistically significant (*P* = 0.624) [21].

Among patients ≤ 64 years of age, those on OTX-101 remained on treatment numerically longer than those on CsA or LFT; median TTD values were 354 days (11.6 months) for OTX-101, 274 days (9.0 months) for CsA, and 261 days (8.6 months) for LFT. Per the log-rank test, differences among cohorts were not statistically significant (OTX-101 vs. CsA, *P* = 0.562; OTX-101 vs. LFT, *P* = 0.958).

In the treatment-naïve subgroup (those who had not received OTX-101, CsA, or LFT prior to index date), patients receiving OTX-101 remained on treatment numerically longer than patients receiving CsA or LFT, though the difference between cohorts was not significant per the log-rank test (OTX-101 vs. CsA, *P* = 0.955; OTX-101 vs. LFT, *P* = 0.210). Median TTD values were 258 days (8.5 months), 229 days (7.5 months), and 251 days (8.2 months) for patients on OTX-101, CsA, and LFT, respectively.

### Probability of treatment discontinuation

After adjusting for patients’ age, Quan-CCI, health insurance program, and eye-related comorbidities, the estimated OR for treatment discontinuation for the CsA cohort relative to the OTX-101 cohort was 1.35 (95% CI, 1.16 to 1.57; *P* < 0.001), indicating that patients receiving CsA were 35% more likely to discontinue treatment than patients receiving OTX-101. The adjusted estimated OR for treatment discontinuation for the LFT cohort relative to the OTX-101 cohort was 0.97 (95% CI, 0.84 to 1.12; *P* = 0.718); thus, the probability of discontinuation was similar for patients receiving LFT and OTX-101.

For patients > 64 years of age receiving OTX-101 or CsA, the adjusted estimated OR for treatment discontinuation relative to patients aged ≤ 64 years was 1.27 (95% CI, 1.07 to 1.51; *P* = 0.007); therefore, older patients were 27% more likely to discontinue treatment than younger patients after adjusting for index treatment, Quan-CCI, eye-related comorbidities, and insurance. In patients receiving OTX-101 or LFT, the adjusted estimated OR for treatment discontinuation in patients > 64 years relative to ≤ 64 years was 1.15 (95% CI, 0.97 to 1.37); however, this difference was not statistically significant (*P* = 0.118) [21].

Compared with patients on OTX-101 or CsA with commercial insurance, the estimated OR for treatment discontinuation for patients receiving these treatments on Medicaid was 0.62 (95% CI, 0.47 to 0.82) after adjusting for index treatment, age, Quan-CCI, and eye‑related comorbidities. This indicates that patients on Medicaid were 38% less likely to discontinue treatment than patients with commercial insurance (*P* = 0.001). In patients receiving OTX-101 or LFT, the adjusted estimated OR for treatment discontinuation for patients on Medicare compared with patients on commercial insurance was 0.79 (95% CI, 0.66 to 0.96), indicating patients on Medicare were 21% less likely to discontinue treatment (*P* = 0.015). Table 3 presents unadjusted and adjusted logistic regression model results for these primary and subgroup analyses.

**Table 3:** Logistic regression model of the probability of treatment discontinuation

	Estimated OR	95% CI	*P*-value
**Unadjusted model**			
CsA (ref OTX-101)	1.41	(1.24 to 1.61)	< 0.001^†^
LFT (ref OTX-101)	1.01	(0.89 to 1.14)	0.917
**Adjusted model**			
CsA (ref OTX-101)	1.35	(1.16 to 1.57)	< 0.001^†^
Age > 64 (ref ≤ 64), years	1.27	(1.07 to 1.51)	0.007^†^
Quan-CCI	0.98	(0.94 to 1.02)	0.343
Eye-related comorbidities (ref no)	0.90	(0.76 to 1.08)	0.262
Insurance (ref commercial)			
Employer	0.87	(0.66 to 1.15)	0.336
Medicaid	0.62	(0.47 to 0.82)	0.001^†^
Medicare	0.94	(0.78 to 1.12)	0.478
LFT (ref OTX-101)	0.97	(0.84 to 1.12)	0.718
Age > 64 (ref ≤ 64), years	1.15	(0.97 to 1.37)	0.118
Quan-CCI	0.99	(0.95 to 1.04)	0.788
Eye-related comorbidities (ref no)	0.85	(0.72 to 1.01)	0.067
Insurance (ref commercial)			
Employer	0.88	(0.69 to 1.10)	0.259
Medicaid	0.79	(0.62 to 1.01)	0.060
Medicare	0.79	(0.66 to 0.96)	0.015^†^

*CI* Confidence interval, *CsA* Cyclosporine ophthalmic emulsion 0.05%, *LFT* Lifitegrast ophthalmic solution 5%, *OR* Odds ratio, *OTX-101* Cyclosporine ophthalmic solution 0.09%, *Quan-CCI* Quan-Charlson Comorbidity Index, *ref* reference

^†^Indicates statistical significance as based on *P*-value < 0.05

### Treatment persistence

**Fig. 7:**
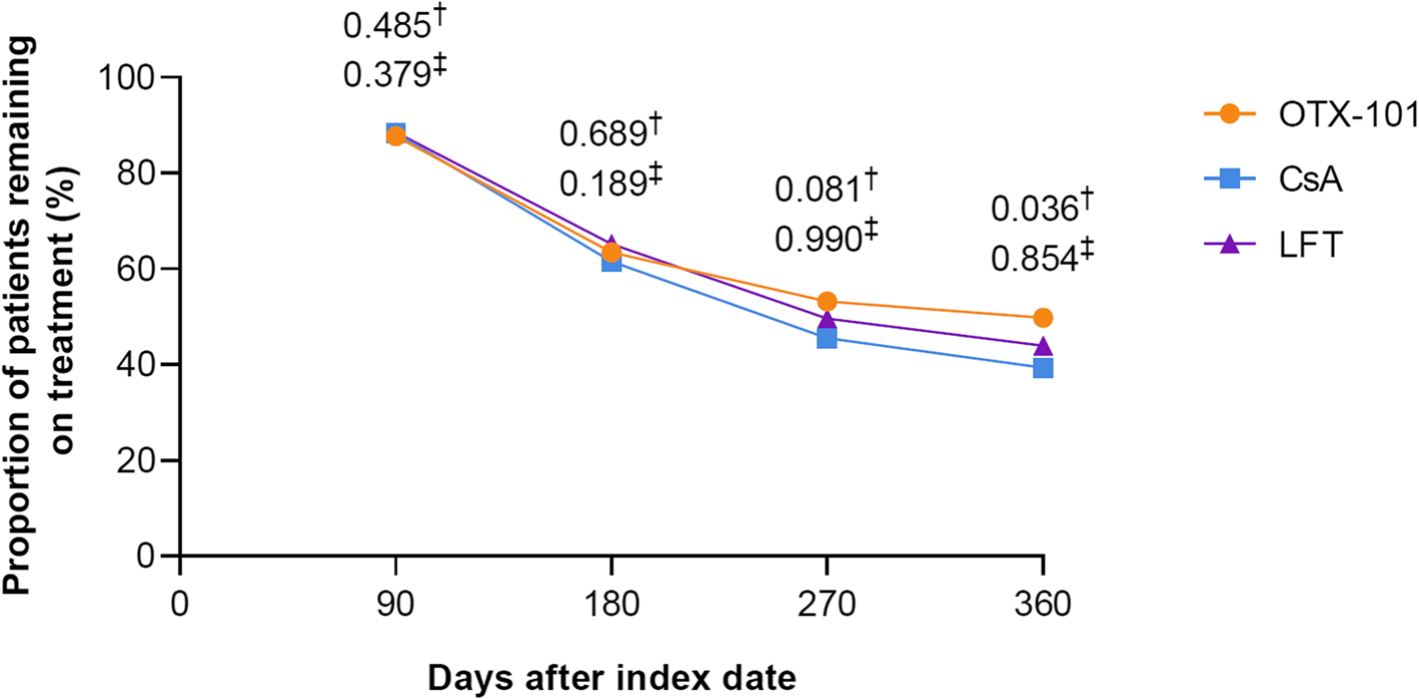
Proportion of patients remaining on index treatment after the index date. ^†^*P*-value for analysis of OTX-101 vs. CsA. The Day 360 *P*-value is statistically significant as based on a significance level of < 0.05. ^‡^*P*-value for analysis of OTX-101 vs. LFT. CsA, cyclosporine ophthalmic emulsion 0.05%; LFT, lifitegrast ophthalmic solution 5%; OTX-101, cyclosporine ophthalmic solution 0.09%

At 360 days after the index date, 49.8% of patients receiving OTX-101 remained on treatment vs. 39.4% of patients receiving CsA (*P* = 0.036) and 44.0% of patients receiving LFT (*P* = 0.854). The percentage of patients receiving OTX-101 who remained on treatment was numerically greater than the percentage of patients who remained on CsA at Days 180 and 270, and the percentage of patients who remained on OTX-101 was numerically greater than the percentage of patients who remained on LFT at Days 270 and 360 (Fig. 7) [21].

## Discussion

This study demonstrated that both median TTD and treatment persistence at 360 days were significantly greater for patients on OTX-101 compared with those on CsA. Patients receiving OTX-101 also tended to remain on treatment longer than patients receiving LFT, though these results were not statistically significant.

Patients receiving CsA were 35% more likely to discontinue treatment than patients on OTX-101 after adjusting for age, Quan-CCI, health insurance program, and eye-related comorbidities, while patients on OTX-101 and LFT had similar likelihoods of discontinuing treatment. In the cohort of patients > 64 years of age, those receiving OTX-101 remained on treatment significantly longer than those on CsA, and time on treatment was similar for patients on OTX-101 and LFT. Additionally, in patients receiving OTX-101 or CsA, older patients were significantly more likely to discontinue treatment than younger patients, while the probability of treatment discontinuation was similar for older and younger patients in those receiving OTX-101 or LFT.

Limited data are available comparing clinical efficacy or treatment patterns in patients with DED receiving OTX-101, CsA, or LFT. No head-to-head clinical trials comparing OTX-101 vs. CsA or OTX-101 vs. LFT have been completed. However, as noted in the OTX-101 Phase 2b/3 clinical trial, OTX-101 was the first DED product to significantly improve both conjunctival staining (*P* = 0.0076) and unanesthetized Schirmer’s test (nominal *P* = 0.0003) compared with vehicle at Day 84, in addition to significantly reducing corneal staining at Day 84 (*P* = 0.0003) [14]. White et al. (2019) analyzed medical insurance claims to compare adherence and discontinuation for CsA vs. LFT in real-world patients with DED [22]. The results indicated that overall adherence was low but was higher for LFT (9.7%) than CsA (5.9%) during the 12-month post-index period [22]. Discontinuation rates within 12 months of treatment initiation were 70.8% for CsA vs. 64.4% for LFT, and median TTD was 89 vs. 29 days for CsA vs. LFT, respectively [22]. These treatment pattern findings are consistent with the results of the current analysis, which found that a higher percentage of patients receiving LFT vs. CsA remained on treatment at 360 days after the index date (44.0% of LFT patients vs. 39.4% of CsA patients). However, the median TTD values in the current analysis (241 days for CsA and 269 days for LFT) were higher than those noted by White et al., perhaps because the current sample selection focused on active users, requiring at least 1 claim for index treatment within 4 months of treatment initiation. Hovanesian et al. (2021) conducted a retrospective, health care provider panel-based chart review study to evaluate real-world treatment patterns in 600 patients with DED treated with LFT [23]. In this study, most patients did not have 12 months of observation, but 238 of the 281 patients with 12 months of observation (84.7%) remained on LFT at 12 months after starting treatment [23]. This value exceeds that of the current analysis, which found a 360-day treatment persistence of 44.0% (1323 of 3008 patients) in the LFT cohort. This inter-study discrepancy in treatment persistence may be attributed to the considerably smaller sample sizes assessed by Hovanesian et al. as compared with the current analysis.

In a cross-sectional survey study of patient satisfaction with CsA vs. LFT, the most common reason for patient dissatisfaction was slow time to onset of effect, which was more frequently observed in patients on CsA than LFT [24]. However, treatment satisfaction after onset of effect was comparable between the 2 agents [24]. The survey study reported that main reasons for treatment switching included doctor recommendation and inability of the current treatment to relieve DED symptoms [24]. Similarly, the retrospective chart review study by Hovanesian et al. (2021) found that the most common reason for LFT treatment discontinuation was insufficient response [23]. As DED is a chronic disease requiring faithful long-term management for good control, and since multiple months of therapy may be required for optimal effect, it is possible that patients tend to switch or discontinue treatment without allowing sufficient time for therapeutic efficacy [7, 24]. Treatment discontinuation may lead to disease progression and worsening of ocular signs and symptoms [22, 25]. Therefore, further research investigating the reasoning behind the high discontinuation rates in real-world DED patients would likely be beneficial for improving treatment persistence [22].

Limitations of this study include the lack of a control group, which may limit contextualization of the results for the 3 treatments. Additionally, since the IDV is a provider-based database, a single patient may be counted multiple times if seen by different providers, though the IDV minimizes this discrepancy with a linking algorithm. The IDV also lacks patient eligibility files, and emergency room visits are not separately flagged, which may affect evidence of clinical activity. Finally, database inaccuracies such as diagnosis miscoding may be present, though these errors are expected to affect all cohorts equally and are not likely to cause biases in the comparative analysis.

## Conclusions

Patients receiving OTX-101 remained on treatment significantly longer and were significantly less likely to discontinue treatment than patients on CsA. Additionally, patients on OTX-101 had numerically longer time on treatment and numerically higher treatment persistence than patients on LFT, though these results were not statistically significant. Older patients receiving OTX-101 remained on treatment significantly longer than those on CsA, while time on treatment was similar for older patients on OTX-101 and LFT. Additionally, older patients on OTX-101 or CsA were more likely than younger patients to discontinue treatment, and the probability of treatment discontinuation was similar for older and younger patients receiving OTX-101 or LFT. These findings provide important real-world evidence regarding treatment patterns for patients with DED receiving OTX-101, CsA, or LFT, which may help to inform the clinical practice of eye care professionals utilizing these therapeutics.

### Supplementary Information

**Additional file 1: Supplemental Table 1.** International Classification of Diseases codes used for patient identification. 

## Data Availability

This study utilized de-identified claims data from the Symphony Health Integrated Dataverse (Symphony Health, Blue Bell, PA), a nationally representative, provider-based claims database including claims submitted to all payer types. The data that support the findings of this study are available from Sun Pharma but restrictions apply to the availability of these data, which were used under license for the current study, and so are not publicly available. Data are however available from the authors upon reasonable request and with permission of Sun Pharma.

## Abbreviations

CI: Confidence interval
CsA: Cyclosporine ophthalmic emulsion 0.05%
DED: Dry eye disease
FDA: US Food and Drug Administration
ICAM-1: Intercellular adhesion molecule-1
ICD: International Classification of Diseases
IDV: Symphony Health Integrated Dataverse
IQR: Interquartile range
IRB: Institutional Review Board
LFA-1: Lymphocyte function-associated antigen-1
LFT: Lifitegrast ophthalmic solution 5%
OR: Odds ratio
OTX-101: Cyclosporine ophthalmic solution 0.09%
Quan-CCI: Quan-Charlson Comorbidity Index
ref: Reference
SD: Standard deviation
TTD: Time to treatment discontinuation
