# Supplementary material for: Real-world treatment patterns of OTX-101 ophthalmic solution, cyclosporine ophthalmic emulsion, and lifitegrast ophthalmic solution in patients with dry eye disease: a retrospective analysis
Source: BMC Ophthalmol. 2023 Nov 2;23:443. doi: 10.1186/s12886-023-03174-y (PMC10621228; doi:10.1186/s12886-023-03174-y)
Supplement: Supplementary file 1 — Additional file 1: Supplemental Table 1. International Classification of Diseases codes used for patient identification. [file 12886_2023_3174_MOESM1_ESM.docx]

**Supplemental Table 1.** International Classification of Diseases codes used for patient identification

| **Condition** | **Code type** | **Code** | **Description** |
| --- | --- | --- | --- |
| Sicca syndrome | ICD-10-CM | M35.00 | Unspecified |
| Sicca syndrome | ICD-10-CM | M35.01 | With keratoconjunctivitis |
| Sicca syndrome | ICD-10-CM | M35.02 | With lung involvement |
| Sicca syndrome | ICD-10-CM | M35.03 | With myopathy |
| Sicca syndrome | ICD-10-CM | M35.04 | With tubulo-interstitial nephropathy |
| Sicca syndrome | ICD-10-CM | M35.09 | With other organ involvement |
| Sicca syndrome | ICD-9-CM | 710.2 | Unspecified |
| Keratoconjunctivitis | ICD-10-CM | H16.22x | Keratoconjunctivitis sicca |
| Keratoconjunctivitis | ICD-10-CM | H16.21x | Exposure keratoconjunctivitis |
| Keratoconjunctivitis | ICD-10-CM | H16.23x | Neurotrophic keratoconjunctivitis |
| Keratoconjunctivitis | ICD-9-CM | 370.33 | Keratoconjunctivitis sicca |
| Keratoconjunctivitis | ICD-9-CM | 370.34 | Exposure keratoconjunctivitis |
| Keratoconjunctivitis | ICD-9-CM | 370.35 | Neurotrophic keratoconjunctivitis |
| Ocular pain | ICD-10-CM | H57.10 | Unspecified eye |
| Ocular pain | ICD-10-CM | H57.11 | Right eye |
| Ocular pain | ICD-10-CM | H57.12 | Left eye |
| Ocular pain | ICD-10-CM | H57.13 | Bilateral |
| Dryness | ICD-10-CM | H11.14x | Conjunctival xerosis |
| Dryness | ICD-10-CM | H04.12x | Tear film insufficiency, unspecified |
| Dryness | ICD-9-CM | 372.53 | Conjunctival xerosis |
| Dryness | ICD-9-CM | 375.15 | Tear film insufficiency, unspecified |
| Sicca syndrome | ICD-10-CM | H16.109 | Superficial keratoconjunctivitis |
| Sicca syndrome | ICD-9-CM | 370.2 | Superficial keratoconjunctivitis |
| Sicca syndrome | ICD-10-CM | H16.149 | Punctate keratitis |
| Sicca syndrome | ICD-9-CM | 370.21 | Punctate keratitis |
